# Designing legislative responses to restrict children’s exposure to unhealthy food and non-alcoholic beverage marketing: a case study analysis of Chile, Canada and the United Kingdom

**DOI:** 10.1186/s12992-022-00865-x

**Published:** 2022-07-23

**Authors:** Fiona Sing, Belinda Reeve, Kathryn Backholer, Sally Mackay, Boyd Swinburn

**Affiliations:** 1 School of Population Health, University of Auckland, Auckland, 1023 New Zealand; 2 The University of Sydney Law School, Camperdown, Australia; 3 Deakin, Global Centre for Preventive Health and Nutrition, Institute for Health Transformation, Geelong, Australia

**Keywords:** Food marketing, Law, Child health, Unhealthy food, Chile, Canada, UK

## Abstract

**Introduction:**

Introducing legislation that restricts companies from exposing children to marketing of unhealthy food and beverage products is both politically and technically difficult. To advance the literature on the technical design of food marketing legislation, and to support governments around the world with legislative development, we aimed to describe the legislative approach from three governments.

**Methods:**

A multiple case study methodology was adopted to describe how three governments approached designing comprehensive food marketing legislation (Chile, Canada and the United Kingdom). A conceptual framework outlining best practice design principles guided our methodological approach to examine how each country designed the technical aspects of their regulatory response, including the regulatory form adopted, the substantive content of the laws, and the implementation and governance mechanisms used. Data from documentary evidence and 15 semi-structured key informant interviews were collected and synthesised using a directed content analysis.

**Results:**

All three countries varied in their legislative design and were therefore considered of variable strength regarding the legislative elements used to protect children from unhealthy food marketing. When compared against the conceptual framework, some elements of best practice design were present, particularly relating to the governance of legislative design and implementation, but the scope of each law (or proposed laws) had limitations. These included: the exclusion of brand marketing; not protecting children up to age 18; focusing solely on child-directed marketing instead of all marketing that children are likely to be exposed to; and not allocating sufficient resources to effectively monitor and enforce the laws. The United Kingdom’s approach to legislation is the most comprehensive and more likely to meet its regulatory objectives.

**Conclusions:**

Our synthesis and analysis of the technical elements of food marketing laws can support governments around the world as they develop their own food marketing restrictions. An analysis of the three approaches illustrates an evolution in the design of food marketing laws over time, as well as the design strengths offered by a legislative approach. Opportunities remain for strengthening legislative responses to protect children from unhealthy food marketing practices.

**Supplementary Information:**

The online version contains supplementary material available at 10.1186/s12992-022-00865-x.

## Introduction

Globally, the consumption of unhealthy food and beverages (ultra-processed foods and beverages, commonly high in salt, sugar, and/or fats) is associated with an increased risk of overweight and obesity, mental health issues, poor dental health and diet-related non-communicable diseases (NCDs) such as heart disease, stroke, diabetes and many cancers [1]. Multi-national food and beverage companies utilise sophisticated, pervasive marketing techniques through multiple channels to increase the sales and consumption of their products in a universal way across the globe [2]. The reach of this marketing is arguably greater now than any time in history due to globalization and rapid increases in digital marketing [2]. Children are particularly vulnerable to such marketing techniques and evidence shows that marketing impacts their preferences, requests, nutrition knowledge and dietary intake [3–7].

Governments across the globe have been called upon repeatedly to implement the World Health Organization Set of Recommendations on the Marketing of Foods and Non-Alcoholic Beverages to Children (WHO Set of Recommendations) [8] and the United Nations Convention on the Rights of the Child (UNCRC) [9], which has been interpreted as requiring children’s protection from exposure to unhealthy food and beverage marketing [10–12]. However, introducing legislation that protects children from exposure to unhealthy food and beverage marketing is both politically and technically difficult [13–16]. Technical challenges relate to which legal vehicle to use, how to engage with relevant stakeholders, how to define key terms such as ‘marketing’ and ‘children’, which mediums and settings to cover, how to effectively capture the full exposure of children to such marketing, whether to legislate all mediums and settings all at once or in a stepwise fashion, who will be liable for breaches and how to monitor and enforce compliance.

A robust body of evidence exists regarding the performance and efficacy of restrictions on unhealthy food marketing to children [17–24]. Self-regulatory approaches are shown to be substantially less effective than mandatory measures at reducing children’s total exposure to unhealthy food marketing and are therefore an inappropriate and inadequate regulatory response to the issue [17, 25–30]. Whilst mandatory regulatory mechanisms are a stronger approach, there has been less systematic examination of their design. Magnusson and Patterson state that laws are a necessary mechanism to combat NCDs and meet global commitments such as the WHO Set of Recommendations and the Global Action Plan on NCDs [31–33]. However, legal capacity is lacking in many countries, and sharing best practices and lessons learned from other governments is imperative for capacity building [32–34]. For example, in Chile, policymakers had no relevant precedent to follow for technical development of the regulation and had to look to tobacco control laws for assistance [35].

To advance the literature on the technical design of food marketing legislation, and to support governments around the world with legislative design, we aimed to describe the legislative approach from three governments (Chile, Canada and United Kingdom (**UK**)). All three countries had introduced legislative Bills, two of which passed, and one of which had been implemented, at the time of writing. We then compared the legal design process and the scope of each law against a set of criteria for best practice design of food marketing restrictions to illustrate the strengths and weaknesses of each countries’ approach.

## Methods

### Case study methodology

A case study methodology was chosen because it allowed for an in-depth investigation of three time-bound cases using multiple sources of data, providing a rich grounding for an analysis of the three cases [36]. The case study approach is familiar in both the fields of law and political science, which this research traverses [36].

The three case study countries were chosen from a scan of global policy databases including the World Cancer Research Fund International (WCRF) NOURISHING database [37], the World Health Organization (WHO) GINA database [38] and a literature review of peer-reviewed and grey literature [11, 28, 39–43]. Included case studies had to meet the following criteria: i) the government attempted to introduce mandatory legislation to restrict unhealthy food and beverage marketing (successful or failed), ii) the legislation (proposed or final) included three or more media or settings through which food marketing was disseminated and iii) there was access to appropriate documentation and iv) recruitment of policymakers and other key informants for interviews was considered to be feasible. The inclusion criteria requiring the legislation to cover three or more media or settings was chosen to capture relatively robust and comprehensive legislative approaches. Access to appropriate documentation meant that we could access the key government documentation, even when the law had not passed, or if time had lapsed. 94 countries were considered, 46 countries of which had a form of mandatory law in place, but the majority of which did not cover three or more media or settings. From a final list of 13, the 3 case countries were chosen based on the access to appropriate documentation and plausible recruitment of policymakers and key informants.

All three case studies had different legislative outcomes, including a regulatory approach that had successfully passed and been implemented (Chile), one that had failed to pass (Canada), and one that had only recently passed into law but the implementation of the law had been delayed for at least 12 months (UK). The cases were time bound by the regulatory design process, beginning with the government’s decision to use a mandatory approach to food marketing regulation, through to the final version of the Bill failing to pass in the Senate (Canada) the time of writing this research (UK) and for Chile, when the accompanying regulations were finalised.

### Conceptual framework

The interview guide and data analysis were informed by a conceptual framework describing best practice criteria for the design and implementation of food marketing regulations (see Table 1). This conceptual framework represents a synthesis of existing public health law frameworks [18, 44],WHO global guidance for regulating unhealthy food marketing [8, 28, 42, 45] and guidance from WCRF [43] and UNICEF [11], as well as peer-reviewed literature [11, 39, 43, 46, 47]. The domains and elements of the framework drew heavily from a public health law framework originally developed by Reeve and Magnusson [18] and later adapted by Jones et al. [44] for analysing and improving the performance of public health regulation. The three domains include regulatory form, regulatory substance, and implementation - each of which represents a distinct stage in the regulatory development/design process. Overarching the three domains is an analysis of the governance mechanisms used in each domain. For the purposes of this research, governance is defined as the relationships, processes, rules, practices and structures put into place to design and implement the law. For example, what legislative mandate was given; who was the lead government agency; how were external stakeholders engaged; and who was given the delegated authority to implement the law and regulations. 

The case study methodology and the underlying conceptual framework allow for an examination of policymakers’ decisions about which legal mechanism to use, how to engage with relevant stakeholders, how to define the meaning of ‘marketing’ and ‘children’, which mediums and settings to include within scope of the law, how to effectively capture the full exposure of children to such marketing, whether to legislate all mediums and settings all at once or in a stepwise fashion, who will be liable for breaches, and how to monitor and enforce compliance. An outline of the framework is provided in Table 1.

**Table 1:** Framework describing best practice technical design and governance of food marketing laws

Domain	Element	Best practice for technical aspects	Best practice for governance
Regulatory Form *The style of regulatory type and mechanism adopted*	Regulatory approach	Legislative ban on marketing practices in scope (not industry-led codes) [11, 28, 48]	A government agency is given the authority to lead the legislative process.
	Legislative vehicle	A legislative vehicle that gives appropriate powers for implementation including enforcement mechanisms. Either a new law or amending existing legislation.	The lead government agency has the jurisdiction to govern the legislative vehicle.
	Legislative objectives/Purpose	Clear, measurable objectives against which the success of regulation can be assessed. With the primary objective to reduce exposure and power of unhealthy food marketing [8, 45]	Government agency sets the objectives it aims to meet with the policy design. Success of meeting objectives assessed by independent body [18].
Regulatory Substance *The design of the substantive content of the law and accompanying regulations*	Definition of children	Up to the age of 18 (unless age of majority reached earlier) [9]	Multi-sectoral consultation *regarding legal design* across multiple areas of government impacted by legislation (e.g. education, communications, trade) Transparent interaction between the government and external actors regarding scope of the law. Appropriate management of conflicts of interest [18, 45].
	Definition of marketing	A broad definition of marketing, such as any form of commercial communication of messages. Intention should be to cover the wide breadth of marketing strategies, including, but not limited to, *advertising (online, broadcast, print media), sponsorship, direct marketing (*e.g. *mail, text), point of sale techniques, product packaging/design and placement and brand marketing* [8, 45].	
	Media, settings and marketing techniques in scope	All media, settings and techniques where children are exposed to unhealthy food marketing, such as *broadcast, online, retail, public spaces and settings, as well as all child-directed marketing techniques and settings* [8, 45]	
	Marketing types in scope	All marketing that children are likely to be exposed to or that has the effect of influencing children’s diets (not limited to marketing that is intended for children) [46]	
	Food classification system to determine what is ‘not permitted’ to be marketed	Informed by evidence-base of foods and non-alcoholic beverages that are considered to be harmful to health and/or aligned with national dietary guidelines [8, 45]	
Implementation *Considerations for implementation design*	Monitoring	Clear and appropriately resourced government-led monitoring system in place free of conflicts of interest [18]	Jurisdictional mandate given to agencies responsible for monitoring
	Enforcement	A wide range of appropriate penalties [18]. Liability - Advertisers (may also consider other entities across marketing chain such as broadcasters, on-demand platforms or social media entities)	Jurisdictional mandate given to implementers to use enforcement mechanisms Ensure domestic legal jurisdiction to hold ‘person’ liable
	Evaluation	Codify periodic evaluation of law into legislation [18].	Independent institution to carry out the evaluation [18] Structured, regular review ensures regulation meeting its objectives. A review framework set during development includes baseline data and, performance indicators and timeframes to evaluate effectiveness [18, 44].

### Data collection

We collated publicly available information on each of the three case studies by systematically searching evidence on official government websites (e.g. parliamentary debates), supplemented by searches of the peer-reviewed literature and grey literature (e.g. WHO or other agency reports). We used key search terms covering: food marketing/advertising; unhealthy food marketing/advertising; food marketing/advertising law; the names of each policy, bill or law (Bill S-228, Food Labelling and Advertising Law, Ley 20.606, Decree 28, 103 and 13, Health and Care Bill ‘advertising restrictions for products high in fat, sugar and salt’) in conjunction with the relevant case country - Chile; Canada; and the UK. The legislation and regulations as well as any guidance provided by the government to the public on the interpretation of the law were identified, along with policy consultation documents provided during the design process. Government documentation from Chile was translated from Spanish to English, including the law and decrees and any Ministry of Health publications. The information from these were triangulated with English documentation from the peer-reviewed literature, other grey literature and with interview data and then verified with our Chilean key informants during the interviews.

The document review was supplemented with 15 semi-structured key informant interviews from Chile (*n* = 3), Canada (*n* = 9) and the United Kingdom (*n* = 8). Key informants included representatives from government (policymakers and politicians), as well as academics and civil society advocates who were heavily involved in designing the law and who could provide details on its technical design. Snowball sampling was used to identify additional key informants when it was established that other areas of expertise were required to construct a fuller picture of the legislative and regulatory design. The data collection, both the document review and interviews, was conducted between April 2021 and April 2022.

As the Chilean legislation had been enacted around 10 years earlier and had been extensively analysed in the literature, fewer interviews were required to reach data saturation. The key informants identified for Chile were also particularly knowledgeable and intimately involved with the design process and were able to provide key documentation that was no longer publicly available, which further enabled saturation to be reached quickly. As the Canadian law had not passed, there was less literature and government documentation available, so a greater number of interviews were required with a broader range of key informants to form a concrete picture of the proposed law. The UK law was going through the legislative process during the research period, therefore key informant interviews were harder to acquire as most government officials could not speak about the Bill. However, the extensive government documentation published during the policy process, including the consultation outcome documents and evidence published to support the Bill and its drafting, significantly aided with the analysis against the conceptual framework.

Ethical clearance was obtained prior to interview recruitment and an informed consent form and plain language statement of the research was given to each participant to sign in advance. A summary document was provided to interviewees ahead of the interview that included a brief outline of the key attributes of the legislative design using the conceptual framework, which the participants were then asked to comment on and revise during or following the interview. The interview questions were guided by the conceptual framework and were intended to garner any additional information required after the document analysis was complete. The interview questions were provided to the participants ahead of time. All interviews were transcribed verbatim. Key informants were provided with an opportunity to review the transcript of the interview to check for accuracy before analysis.

### Data analysis

A directed content analysis was carried out [49], which involved extracting and synthesising the documentary data and the semi-structured interview data according to the domains and elements of our guiding conceptual framework. This data was then compared and contrasted with best practice design from our framework and across case-studies to understand the strengths and weaknesses of the legal design for each case study. The rationale for the legislative approach selected, the role and influence of actors and relevant external events were also described in order to provide context to the development of each country’s legislative scheme. One author (FS) conducted the interviews and extracted and analysed the data. All extracted data was reviewed with all authors and any discrepancies in understanding were discussed and resolved.

## Results

### Regulatory form

#### Regulatory approach and legislative vehicle

As per the case study selection criteria, all three countries designed a mandatory legislative approach to restricting unhealthy food marketing. In Chile, child obesity rates were considered to be at unacceptable levels and Senator Guido, a medical doctor, campaigned over many years to introduce new, dedicated and comprehensive legislation to address the issue with the support of a leading academic [50, 51]. Key informants stated that the pre-existing self-regulatory system had failed to address the issue adequately. The Government gave responsibility for designing and implementing the marketing restrictions to the Ministry of Health [35]. Law No. 20.606 on the Nutrient Composition of Food and its Advertising (the Food Law) introduced multiple policies, including marketing controls, school food provision and front of package warning labels [52]. The Law also amended Sanitary regulations that related to these areas [50, 52, 53]. The mandate and infrastructure for Sanitary regulations and food policy sat with the Ministry of Health [50, 52].

In Canada, a self-regulatory approach had been in place since 2007, with multiple academic publications showing its ineffectiveness [25, 54, 55]. The Liberal Party of Canada campaigned on the issue of improving health through nutrition policies, and after successfully entering government, included protecting children from unhealthy food and beverage marketing in the Prime Minister’s ‘mandate letters’, which outlined what the Minster of Health must accomplish [56]. Simultaneously, Conservative Senator Nancy Greene Raine introduced a Senate Bill to protect children from unhealthy food and beverage marketing (the Child Health Protection Act, also known as Bill S-228) [57]. The Government decided not to introduce a Government Bill but to follow and support the Child Health Protection Act through the parliamentary process as it was already in motion and had the same objectives.

As the subject was both a Minister of Health issue because of the mandate letters but also a health-related Senator’s Bill, the Minster of Health, and therefore Health Canada (the Canadian Ministry of Health) were the lead government actors. As such, two different departments within Health Canada (primarily the Office of Nutrition Policy and Promotion and later the Food Directorate) were given authority to design the marketing restrictions outlined in Bill S-228. It was decided that the Food and Drug Act would be the enabling legislation that the Bill would amend, and the Food Regulations would contain the specific regulatory requirements. This was because the Minister of Health had the governing jurisdiction over that legislation but also the existing infrastructure around nutrient limits and the existing broad definition of ‘advertising’ in the Act was an appropriate fit for the intent of the Bill. Other legislative avenues were explored, including the Consumer Protection legislation, used in the Quebec region to restrict unhealthy marketing to children. However, that area of law was governed by a different government Ministry (Industry Canada) and is not enforced at the federal level, rather at a regional level, and so could not be utilised by the Government. Ultimately, while Bill S-228 passed through the Senate and House of Commons once, when it was returned to the Senate, it did not pass its final reading before an election was called and therefore did not pass into law.

The UK also had co-regulatory and self-regulatory marketing restrictions in place prior to the development of the new laws, which had been evaluated by the academic community as having limited effect [22, 24, 58]. The Conservative Government had introduced a Childhood Obesity Strategy with a comprehensive suite of policy interventions to address obesity, which included introducing stronger restrictions on food and beverage marketing [59]. The Department of Health and Social Care partnered with the Department of Digital, Culture, Media and Sport to develop the marketing policy, particularly the elements relating to the online environment. Amendments relating to food marketing restrictions (broadcast and digital) were incorporated into the larger Health and Care Bill to work through the political process [60]. The relevant clauses of the Health and Care Bill stated that the Communications Act 2003 was to be amended to reflect the policy options for broadcast and online advertising chosen by the Government after public consultation. A further policy introduced a ban for all volume-based price and product placement promotion marketing techniques in retail settings which were introduced under secondary legislation entitled The Food (Promotion and Placement) (England) Regulations 2021 [61].

In each case, a legislative response followed the documented failure of self-regulation. Each government took a different approach to the legislative vehicle they used to introduce the law: Chile introduced new dedicated legislation; Canada amended existing food legislation; and the UK their communications legislation. All three case countries chose not to take a stepwise approach that would introduce the laws incrementally but instead adopted a comprehensive package of restrictions. In all three cases, a lead government agency was given the appropriate mandate to lead policy design using a legislative vehicle that they had jurisdiction over.

#### Policy objectives

Canada and the UK both stated that reducing the exposure of children to unhealthy food and beverage marketing (framed as leading to the overconsumption of such foods) was a primary objective of the legislation [62, 63]. Canada specifically mentioned the link between advertising and food preferences and choices, resulting in overconsumption which can lead to poorer health outcomes [63]. This indicated a longer-term goal of reducing diet-related diseases. The UK also stated that the law should drive reformulation of products by brands; be proportionate and targeted to the products of most concern to childhood obesity and limit the advertising children see; and be easily understood by parents [62]. Chile had three objectives for the comprehensive package of laws it introduced: first, protecting children; second, promoting informed food selection; and third, reducing consumption of high fat, sugar, salt food [64].

The policy objectives chosen align with best practice guidance, particularly Canada and the UK, both of which focused on reducing children’s exposure to unhealthy food and beverage marketing. Chile had wider objectives given its law dealt with other policy areas beyond marketing and it was the only country of the three to include protecting children as a stated policy objective.

### Regulatory substance

The design of the substantive content of the law and accompanying regulations entails an assessment of a wide range of technical points. Table 1 outlines the relevant considerations when designing the regulatory substance of food marketing laws by outlining the best practice technical features and good governance principles. Of note is that in order to meet the regulatory objective of reducing the power and exposure of children to unhealthy food marketing, key terms and conditions must be clearly defined and the regulatory rules must be sufficiently expansive [18]. Table 2 provides an overview of the key marketing types regulated in each country. A more detailed summary of the key substantive elements of each law is outlined in Table 3 followed by a discussion of whether each country met best practice design elements in its approach.

**Table 2:** Summary of marketing media, settings and packaging covered

	Chile (child-directed marketing)	Canada (child-directed marketing)	UK
Broadcast media	☒	☒	☒
Radio	☒	☒	☐
Online environment	☒	☒	☒
School settings	☒	☒	☐
Billboards, flyers, banners, posters	☒	☒	☐
Retail settings (point of sale OR promotions)	☒	☐	☒
Food packaging	☒	☐	☐
Public settings	☒	☒	☐

**Table 3:** Regulatory Substance - the scope of three legislative responses

Country	Legislation	Age protected	Nutrient profile used	Types of marketing, settings, media, and techniques covered
Chile [50–52, 65–69]	Law No. 20.606 on the Nutrient Composition of Food and its Advertising amending Supreme Decree No. 977 of 1996, to amend the Food Sanitary Regulations The law passed June 2012 (after five years of debate in Congress and the Senate) Accompanying regulations (Decrees) design process from June 2012–June 2015 Commencement date: June 2016.	Up to 14	The nutrient profile model applied to packaged and non-packaged food and increased in scope in three increments (date of implementation of law; 24 months after implementation; 36 months after implementation. *Solid food* Energy kcal/100 g (350 > 300 > 275) Sodium mg/100 g (800 > 500 > 400) Total Sugars g/100 g (22.5 > 15 > 10) Saturated fat g/100 g (6 > 5 > 4) *Beverages* Energy kcal/100 ml (100 > 80 > 70) Sodium mg/100 ml (100 > 100 > 100) Total Sugars g/100 ml (6 > 5 > 5) Saturated fat g/100 ml (3 > 3 > 3)	Includes television, radio, magazines, digital marketing, billboards, flyers, shop windows, food packages, and point-of-sale boards as well as preschools, elementary schools, and public spaces. Includes marketing techniques such as free food or toys, games, and prizes associated with food and beverage products.
Canada [57, 63, 70]	Child Health Protection Bill (Bill S-228) amending the Food and Drug Act Introduced in Senate 2016 Failed final assent in 2019	Up to 13	Added saturated fat - A total of 2 g saturated fatty acid (SFA) per reference amount (RA) or serving of stated size, whichever is the greater* and ≤ 15% energy from the SFA. Added sodium - 140 mg per RA or serving of stated size whichever is the greater or 140 mg per 50 g of the product if the RA is ≤30 g or 30 mL Sugar - 5 g per RA or serving of stated size whichever is the greater or 5 g per 50 g of the product if the RA is ≤30 g or 30 mL The thresholds are equivalent to the “low in” thresholds for the nutrient content claims “low in saturated fat”, “low in sodium” and “low in sugars”.	All formats and media that are used to communicate or broadcast advertising, including radio and television; the internet; mobile phones; printed materials; promotional items; billboards, banners and posters. Also intended to be included in the definition were sponsorship, but not child sports sponsorship, and point-of-sale advertising [63].
UK [60–62, 71]	Schedule 17 of Health and Care Bill amending Communications Act 2003 Government announced policy June 2018 Public consultation Jan 2019- Dec 2020 Introduced into parliamentary process – June 2021 Law passed April 2022. The Food (Promotion and Placement) (England) Regulations 2021	Not relevant as legislation is not focused on marketing directed to children	‘Less healthy’ foods that meet a threshold of energy, saturated fat, total sugars and sodium (after consideration of the fruit, vegetable, nuts, fibre and protein components). Scores are allocated against a 10 point scale ranging from ≤335 - > 3350 kJ (energy); ≤ 1 g - > 10 g (sat fat); ≤ 4.5 - > 45 g (sugar); ≤ 90 - > 900 mg (sodium); ≤ 40 - > 80 (fruit, veg, nuts); ≤ 0.7 - > 3.5 g (NSP fibre); ≤ 0.9 - > 4.7 g (AOAC fibre); ≤ 1.6 - > 8.0 g (protein)	Broadcast television and on-demand programmes, Online advertising Price and location promotions in retail settings
How the different marketing settings, media and techniques are regulated				
Chile	*Broadcast television and cinema*– All marketing of unhealthy food and beverages banned from 6 am to 10 pm. *All other advertising of unhealthy food and beverages* must not be ‘targeted at children’. - ‘self-identified’ as targeting children (such as advertising during children’s programming or a child’s event) or -- children under 14 make up more than 20% of the intended audience. Or - has child appealing characteristics including child characters; figures; animations; cartoons; toys; children’s music; the presence of people or animals; statements or arguments about the product or its effects; children’s voices; language; expressions; situations that represent their daily life for example school or games			
Canada	-Advertising placed in a setting intended for children or in a medium where children made up 15% of the audience. Also included were settings (places, events, or activities) where children are ‘generally or frequently in attendance’, with the nature and purpose of the event or activity to be considered. -Advertising targeted or appealing to children through its design, characteristics or use of techniques such as: Child-appealing subjects or themes; wording, lettering, colours, special effects, 3D animation, music, songs or jingles; language; shape, colour, design; characters like heroes, cartoon characters and images, animals and imaginary, fantasy or virtual creature; Celebrity endorsements, tie-ins and cross-promotions; Situations that play on the parent-child relationship in an insistent or idealized manner; and Incentives or offers such as toys, prizes, giveaways, discounts, games etc. [63]			
UK	*Broadcast and on-demand television:* Full ban on all advertising of ‘less healthy’ food and drink between 5.30 am and 9 pm *Online:* All paid for advertisements of ‘less healthy’ food and drink is banned, if advertisement payments are made from a person who carries on business in the UK and the advertisement is intended principally for a UK audience. ‘Paid for’ includes advertising where the payment is in the form of money or through other non-monetary benefits (e.g products given to an influencer in return for social media review or promotion) *Retail:* Location restrictions apply to store entrances, aisle ends and checkouts and their online equivalents (entry pages, landing pages, checkout pages).Volume price restrictions prohibit offering promotions such as “buy-one-get-one-free” or “3 for 2” offers on certain products.			

#### Definition of child

In both Chile and Canada, the definition of child was debated and amended, in Chile from ‘up to the age of 18’ to 14 [52] and in Canada from ‘persons who are under the age of 17’ to 13 [57, 70]. As the approach in the UK was not centered on children and did not require an assessment of whether advertising was ‘child-directed’, the legislation does not define ‘children’. Key informants stated that the age in Chile was settled on because other existing laws and regulations in Chile used this age threshold to define children, which made it hard to argue for a higher age. In both Chile and Canada, actors opposed to the scope of the laws argued that, technically, it would be hard to discern between marketing techniques that appeal to a 16 or 17 year olds compared to an 18 year old or to other young adults. Ultimately, best practice design, which would protect children up to 18, was not reached in those two cases. For the UK, the full online ban and retail settings ban would have the effect of protecting children up to 18, but the 9 pm broadcast watershed would only protect those children who do not view television beyond 9 pm.

#### Definition of marketing

Chile and Canada both chose broad definitions of advertising that covered all forms of promotion. Chile’s Food Law defined advertising as “all forms of promotion, communication, recommendation, propaganda, information or action aimed to promote the consumption of a certain product.” [52] Canada’s Bill S-228 defined advertising as “any representation by any means whatever for the purpose of promoting directly or indirectly the sale or disposal of any food” [57]. Chile’s definition centred on any mechanism that aimed to promote consumption of a certain product whereas Canada’s definition focused on the purpose of the promotion being the sale, or disposal, of a product. The UK did not define marketing or advertising in its broader sense but did define all online marketing communications as those “which have the effect of promoting identifiable HFSS products” It is assumed the government didn’t define advertising or marketing because it was focusing on three specific forms: online, tv and retail settings.

All countries perceived brand advertising (where a brand is marketed without featuring a specific food or beverage product) and sponsorship as key challenges when deciding on the scope of restrictions. None of the three countries included brand advertising within scope of the law, despite growing evidence that it impacts children’s food preference and selection [72–74]. Chile did however regulate brand icons considered to be ‘child-directed’ but, in general, brands could advertise if they didn’t refer to a regulated product. The UK exempted brand advertising as it was perceived that it could potentially disincentivise brands from reformulating their products if a negative stigma was attached to their brand [75].

Sponsorship by food or beverage companies was included in the scope for all three laws, but only when a food or beverage product was present in the advertisement. For example, Canada would have permitted the sponsorship recipient to advertise the brand or name of the sponsor company, but this could not include reference to a restricted food product or any specific brand elements associated with the food, or otherwise be designed in a way that targets or attracts children [63]. In a late stage of policy development and after receiving political push back, Canada changed the scope of the law so that food companies could sponsor events and child athletes in settings and media otherwise restricted, as long as sponsorship did not depict unhealthy foods, use child-appealing techniques, characters, images, celebrities or mascots, offer promotions or use language to incite a child to purchase the food [63].

Therefore, while a broad definition of advertising or marketing was adopted by all countries, there were still loopholes in relation to sponsorship and brand advertising.

#### Media, settings and marketing techniques in scope

The media, settings and techniques covered by the laws varied across the three countries. All three regulated broadcast and online media as well as point of sale techniques in retail settings. Canada and Chile regulated schools and other places children gather, as well as billboards, shop windows and flyers. Chile also extended the law to public spaces, not just those where children gather, and included front-of-pack promotions, although for all these media and settings only marketing content considered to be directed to children was included in scope.

The number of media and settings was less for the UK, which focused on broadcast and on-demand television, the online environment and retail settings. These media and settings were selected based on research showing high levels of food marketing exposure in these settings and media, and because the Department of Health and Social Care had a mandate over these areas. School settings were excluded as the responsibility for regulating the school environment fell under the Department of Education. Broadcast radio, print, outdoor settings, direct marketing and cinema were not included within the scope. However, despite the fewer media and settings included in scope, the law captures more unhealthy food marketing within these media and settings compared to Chile and Canada as it is not limited to marketing that is considered to be ‘child-directed’.

Both the UK and Chile exempted small and medium size businesses from their law. The UK based this on employee numbers or shop size (for retail regulations) with exemptions ongoing, while Chile based it on businesses’ income, and by 2022 this exemption was removed. In relation to the UK’s online marketing ban, companies were still able to advertise on their own website or their own social media pages and to other businesses. The law also only applied to ‘paid’ advertising, meaning that any advertising that had been placed through a form of payment was in scope of the law and user-generated or user-shared advertising content was exempt. The online restrictions only apply to companies who carry out business in the UK or to advertisements placed by overseas companies that are intended to be accessed primarily by people in the UK [60].

Although all three countries regulated a range of media, settings, and techniques, there were still gaps or loopholes in the approach taken in each country.

#### Marketing types in scope

Both Chile and Canada limited the scope of the law to marketing that is intended to appeal to children, rather than including all marketing that children are exposed to, irrespective of the intended audience. Initially, both the Chilean and Canadian governments used a combination of audience thresholds (at least 20 and 15% of children in the audience, respectively) and creative content (e.g use of cartoon characters or celebrity endorsements) to determine whether the advertisements were intended for children or not. After the Chilean law was implemented, the Government changed the broadcast element of the law in June 2018, to a television watershed ban on all unhealthy food and beverage advertising between 6 am and 10 pm because the effectiveness of the ‘child-directed’ broadcast law was found to be limited [66, 67].

In Canada, advertising of certain products was to be prohibited in settings where children were generally or frequently in attendance, which could be discerned by the nature or purpose of the event or activity held at the setting. However, where children made up less than 15% of the audience, or if the setting had a mixed audience, then the creative content of the advertisement and the promotional techniques used were assessed to determine if it was directed at children [63].

Instead of taking a child-directed approach, the UK Government aimed to reduce children’s exposure to all unhealthy food and beverage marketing. The definition of children was therefore not included in the legislation nor was any explanation of what is ‘targeted’ or ‘directed’ at children. A time-based, setting-based or media-based design was utilised to capture more of children’s full exposure to unhealthy food and beverage marketing, meaning each of the three areas were regulated in a different way that was appropriate to its characteristics. A time-based ban suited the linear broadcast medium but not the online setting, where a time-based design was deemed to be impossible to enforce. Instead, the Government introduced a full ban on all paid-for unhealthy food marketing in online media, and a blanket ban for all volume-based price and product placement promotion marketing techniques in retail settings, as child-directed thresholds or definitions were thought to be inadequate to meet the policy objective of reducing children’s exposure to unhealthy food marketing.

The approach taken by the UK and the change in the broadcast provision by Chile to a watershed design aligns with best practice as it represents a move away from banning ‘child-directed’ marketing to regulatory design that captures children’s full exposure to unhealthy food marketing, regardless of that advertising’s target audience.

#### Food classification system

While the nutrient profile models used by the three governments have their differences, they are comparable in the following ways. Sugar, fat and salt/sodium were the nutrients of concern measured based on a per gram or millilitre measurement (UK and Chile) and per serving (Canada). Each country tailored the threshold assessments to their national context and either adapted or borrowed the thresholds from other regulations in their jurisdiction. For example, the Canadian thresholds were taken from their nutrient content claim regulation so the food and beverage industry were familiar with them. The UK used the Food Standards Agency 2004–2005 Nutrient Profile Model to determine which foods would be covered by both the broadcast and online law and the retail regulations [71, 75]. In Chile, extensive research and consultation went into the developing the nutrient thresholds for the entire Food Law [35, 51, 67, 76]. In line with best practice design, all three countries utilised food classification systems informed by an appropriate evidence-base and aligned with their national dietary guidelines.

#### Governance of policy design

##### Multi-sectoral consultation

In Canada and Chile, the law had to be approved by the Prime Minister and President’s Office, respectively, which required a consensus amongst key government sectors, therefore, a degree of multi-sectoral consultation took place. This was particularly important with other government agencies and Ministers where the scope of the law could impact on their portfolios, such as education, communications, agriculture and trade. In the UK, the Department of Health and Social Care co-designed the law with the Department of Digital, Culture, Media and Sport due to the crossover of the law with each of their legal jurisdictions, whereas the Health Ministries and agencies in Chile and Canada were solely responsible for the design of the relevant laws.

##### Interaction between the government and external actors

Key informants considered that the key policy development processes in Chile and Canada were led strongly by the Ministries of Health, and in the most part, protected from direct industry influence. During the Decree development, the Chilean Ministry of Health convened two expert committees: one to work on the nutrient thresholds and one to work on other aspects of the law, including the advertising restrictions. Their role was to make decisions on technical aspects of the law [35]. The technical committees were made up of experts and academics without any representation from the food and beverage industry. The Chilean Ministry of Health did meet with industry regularly throughout the drafting process, but key informants felt they were not under pressure to take on board any feedback received if it didn’t fit with the Government’s intentions, which they considered to be strongly protective of public health. During the development of the Canadian Bill, due to its governance rules, Health Canada’s Office of Nutrition Policy and Promotion did not meet with any external stakeholders that may have had conflicts of interest with the policy in development. In addition, the Government adopted an openness and transparency policy, which meant that a copy of all communications received or meeting minutes held with external parties about the Bill were published online. Key informants believed this helped reduce the amount of lobbying the policymakers were subject to. However, they reported that, in line with their governance procedures, meetings with external stakeholders, including the food and beverage industry, did take place when the regulations were being developed by the Food Directorate branch of Health Canada. Health Canada’s Office of Nutrition Policy and Promotion engaged with the ‘Stop Marketing to Kids’ health coalition and leading academic experts on food marketing throughout the policy development to gather the most up to date evidence and guidance on best practice policy design.

Given the proximity of the research to the UK’s process of legislative development, there is less information available about the governance structures in place, including regarding engagement with external actors. Key informants reported that the Department of Health and Social Care engaged with the Obesity Health Alliance coalition of public health advocates intermittently throughout the policy development process. Academic experts were predominantly engaged with by the Department of Health and Social Care through the Obesity Health Alliance relationship however the Department did rely on academic expertise through the Government’s Obesity Policy Research Unit, in particular the Institute for Fiscal Studies.

##### Public consultation

All three governments consulted the public on the proposed policy. Chile’s Ministry of Health ran participatory dialogues, citizen forums and engagement with school communities across the regions of Chile [35]. Civil society also played a role in aiding the government with the consultation process using social media, and the food and beverage industry were invited to engage with the consultation process [35]. Health Canada ran public consultations on the scope of the Canadian Bill. Later a draft ‘Guide to the Application of the Child Health Protection Act (Bill S-228)’ was published for public comments [63]. While these consultations did help the respective governments with policy design, it is unclear exactly which elements were ultimately influenced by consultation responses.

The UK Department of Health and Social Care initially ran public consultations for a time-based watershed approach to both broadcast and online advertising, as well as a separate consultation on the retail policy. Subsequently, a second public consultation on a full online ban was required because it was determined that a watershed approach would not work for the online environment due to a lack of transparency and independent data available on advertisements and an inability for online technical controls to protect children from exposure effectively [62].

Extensive public consultation enhanced the transparency and accountability of legislative drafting processes and created opportunities for the public to have a say on what the legislation would look like. At the same time, the fact that legislative drafting processes were led by government departments, who each had varying degrees of governance structures in place, meant that the Government could decide how it chose to engage with industry during the legislative development process.

### Implementation

Accounting for how the law will be implemented is an essential part of the policy design process to ensure the law can be enforced and that it will effectively meet its objectives [43]. The focus of the Implementation Domain remains on the design phase of the law and how policymakers plan for monitoring, enforcement and evaluation. It does not assess whether those implementation measures have been adequately established in the case study jurisdictions.

As the Canadian Bill S-228 was not passed and the UK legislation was recently passed at the time the research was conducted with the implementation of the UK law then delayed, the details on monitoring, enforcement and evaluation were not available. However, some information could be gathered from the interviews and the publicly available documents about the intentions of both governments. Table 4 outlines how monitoring, enforcement, and evaluation was planned for in each jurisdiction.

**Table 4:** Implementation of the law – key elements

	Monitoring	Enforcement	Evaluation
Chile	The Law delegated responsibility for monitoring to the Ministry of Health. The Ministry then trained and delegated the Regional Ministry of Health Officers (the Regional Health Ministerial Secretary) to monitor compliance. Other agencies were also used to help monitor and enforce the Law [53].	Enforcement of the law was delayed one year to allow industry to prepare. First breach of law – warnings issued Subsequent breaches - withdrawal or removal of the advertisement or a legal ‘administrative summario’ was issued, which is accompanied by a fine [68]. Liability - Advertisers	The Ministry of Health carried out an evaluation of the implementation process of the law after six months. A second evaluation took place one year after the law was in place, with the results of the evaluation being made publicly available [53].
Canada	As the Bill did not pass, the mechanics of how to monitor the regulations were not established. Resources were earmarked to be allocated to the relevant government agencies to aid with data collection, analysis and annual reporting on children’s exposure and emerging advertising techniques. Tactics, settings and techniques not covered by the regulatory framework were to be monitored to see whether the scope of the law needed to be amended.	The enforcement powers had not been decided, but under the Food and Drug Act there are a range of enforcement powers that the Government would have carefully selected from in relation to the jurisdiction they had including to inspect, seize, and forfeit contravening items as well as the power to remedy any contravention of the Act. As the Food and Drug Act is a criminal law, any contravention of the Act could result in a conviction which could lead to a fine or imprisonment term. Liability - Advertisers	A five-year review was planned at the outset to assess the law overall, but to also determine whether unregulated areas needed to be incorporated into the scope of the law.
United Kingdom [60–62, 75]	The detail of how the law will be monitored were to be outlined in forthcoming regulations that are subject to delay. The Office of Communications (**OFCOM**) is the appointed statutory regulator and it is anticipated they will delegate this authority to the Advertising Standards Authority (**ASA**). The Law does give the regulator power to request information to allow it to carry out its functions such as monitoring for enforcement.	*Broadcast and online:* The law stipulates two enforcement provisions: an enforcement notification or a fine. The maximum penalty is the greater of 5% of the turnover of the person’s relevant business for the relevant period, and £250,000. Liability - Broadcasters and on-demand platform services under UK jurisdiction (including video-on-demand) will be liable for breaches of the TV watershed. For non-UK on-demand platform services, the advertiser will be liable for breaches of the online restriction on these platforms. *Retail settings:* Regional enforcement officers can issue an improvement notice before any penalty can be levied, affording businesses an opportunity to take steps to comply with the requirements. Non-compliance with an improvement notice is an offence; in such cases enforcement officers may impose a fixed monetary penalty of £2500 using powers under the Regulatory Enforcement and Sanctions Act 2008. This is intended to provide food authorities with a proportionate alternative means of enforcement to criminal prosecution under the Food Safety Act 1990.	The law states it will be reviewed and evaluated within five years post-implementation.

#### Jurisdictional mandate given to implementers

Chile and the UK gave clear, appropriate jurisdiction to the relevant government departments and agencies to monitor and enforce the laws. The consideration of who had governing authority for monitoring and implementing the law had not been addressed in Canada as the law did not progress. In addition, in most cases, the advertiser was liable for compliance with the legislation, and the relevant government department/agency had legal authority to hold such ‘persons’ liable. This was also the case in the UK where the broadcaster and the on-demand platforms were liable under the relevant legislation.

#### Monitoring for compliance

For the UK and Chile, the task of designing the implementation of the law was delegated to an agency – the Ministry of Health (Chile) and OFCOM (UK) who will likely delegate this role to the ASA. Transparency around how the ASA will interpret the law and its role as the implementer is not apparent. The relevant law in all three cases did not stipulate how monitoring would take place, as that is likely to be dealt with in regulations, which in the case of UK and Canada were not developed. In Chile, the Decree did not outline the monitoring steps, but a manual with extensive guidance was provided to the implementers of the law and training workshops. The UK law does provide regulators with the power to request information that will enable them to carry out their functions under law, a provision that could enable the ASA to require information from advertisers to more accurately monitor their behaviours. The laws in each jurisdiction also failed to contain provisions on how the implementation and enforcement of each law would be resourced. In Chile, key informants stated the system required significantly more financial and human resourcing to improve its efficacy, something that could have been established at the outset of the legal design, but was not. The absence of legislative provisions on resourcing appears to be a key limit to food marketing laws in all three jurisdictions.

#### Enforcement

While a variety of penalties existed in the UK and Chile (Canada had not reached this stage), the effectiveness of those penalties was questioned by the key informants, who stated, for example, that fines were not always high enough to deter non-compliance. More effective penalties were explained as those that would remove an offending entire marketing campaign across all mediums, or reduce a company’s ability to market certain products if they breached the law. Some key informants stated that a pre-vetting function could also increase compliance which had previously existed in the UK, however it was unclear whether this system would continue under the new regime. Accordingly, it was perceived that there was scope to extend or strengthen the enforcement regime that accompanied legislation in each country.

#### Evaluation

In all countries evaluation of the law was considered in the design phase and often included in the legislation or the accompanying government documents. However, it was not stipulated that an independent institution should carry out the evaluation. Also absent was a clear mandate to allocate resources to measure benchmark metrics and ongoing monitoring of the impact and unintended consequences of the laws. Both the UK and Canada had a five-year review process, while Chile started with 6 months, with other annual reviews planned. For the purposes of evaluation, Health Canada ensured resources were allocated for monitoring the law, and when elements of the policy were changed during the policy process, such as the age threshold, they extended the monitoring mandate to cover measures, settings and techniques excluded from the law. This would have enabled them to argue for extending the scope of the law if necessary. For example, teenagers’ exposure to unhealthy food marketing would be monitored to assess whether it had increased since the law was introduced. This meant the evaluation could provide more concrete recommendations on extending the scope of the law if there were unintended consequences, such as a shift in marketing spends to target adolescents. However, the legislation did not stipulate that an independent institution was to carry out the evaluation.

## Discussion

This case study analysis demonstrated variation in the design of food marketing laws in Chile, Canada, and the UK, and therefore variation in the ability of such laws to protect children from unhealthy food marketing in these three countries. While some elements of best practice design were present, particularly relating to the governance of design processes, the scope of each law (or proposed law) had limitations. The key weaknesses identified included: the exclusion of brand marketing; not protecting children up to 18; focusing solely on child-directed marketing instead of all marketing that children are exposed to; and not codifying the provision of resources to effectively implement the laws. These findings align with other research demonstrating that while legislation has many strengths compared to self-regulation, legislative regimes often contain significant loopholes that potentially limit their effectiveness [18, 28, 30, 39, 40].

The three countries included in our analysis represent jurisdictions from three different global regions (Latin America, North America and Europe), and while each has different political, economic and cultural systems, the design approach each used in its respective legislation was similar. In addition, the three case studies represent a range in time, with Chile’s law being designed first, based heavily on tobacco control restrictions, and Canada and the United Kingdom following from Chile’s experience.

The three cases illustrate the evolution of the legislative design in response to the issue of food marketing. Comparing the three cases shows a move away by some countries from the ‘child-directed’ approach that limited the Chilean and Canadian response towards the UK’s approach of reducing children’s exposure to all unhealthy food marketing, regardless of its target audience. This is a much stronger approach, as children are exposed to marketing in all aspects of their lives, and this exposure has a demonstrable impact on their preferences, requests, purchases and consumption, even if the advertisements they see are not specifically targeted to them [72, 74]. This design also meant that the arduous and hard to enforce ‘child-directed’ mechanisms became obsolete, along with it, the arguments about what age of child to protect. Defining the age of a child is a common battleground, as seen in both the Chilean and Canadian examples where the age threshold was reduced [35, 43, 50, 51, 53, 76, 77]. The age threshold could be challenged based on the argument that it was difficult to understand what techniques or characteristics would be directed to children aged 16 or 17 compared to an 18-year-old or older; or that the age thresholds in other pieces of legislation were lower. In contrast, the UK did not require a definition of child in their legislation and a list of techniques or characteristics of what could be considered appealing to children is not required for the effective implementation of the law. This approach creates a more comprehensive law in terms of the level of exposure that it restricts, which can more effectively meet its objectives, if exemptions are not carved out. It also makes the enforcement more straightforward, as all advertising of unhealthy products across broadcast, on-demand, online and retail settings (by price and location) are prohibited.

However, the UK’s approach is not without its limitations. All countries grappled with and failed to address the pressing issue of brand marketing, including the UK. The online environment is also an incredibly difficult area to regulate, and while the UK law goes some way towards adequately regulating digital marketing, only paid advertising is included, when earned and owned marketing, such as user-generated content or shared content, make up a large component of children’s exposure to online food marketing [78].

Despite these opportunities for improvement, the case study countries still represent the most comprehensive examples of food marketing regulatory responses globally. The laws cover multiple media, settings, and techniques (broadcast, online, settings where children gather, outdoor advertising, point of sale advertising etc) and use a scientifically based nutrient profile model to categorise which foods are in scope of the law. In terms of governance processes, the design and implementation of these laws was led by a government department and accompanied by processes of expert and community consultation and engagement, with minimum direct industry involvement. This is in strong contrast to industry self-regulation of unhealthy food marketing to children, which is developed by industry bodies, often without community consultation, and lacks transparency and accountability to the public and other external stakeholders [18, 19].

A key area of legal design that does need more attention is the enforcement provisions for each law. While this research does not assess the implementation of the laws in practice, and in reality, only Chile’s law has been implemented, some of the data on the proposed design of implementation shed light on how improvements could be made. While the laws delegate authority for implementation clearly, the findings from key informant interviews were that a lack of resources hampered the effective monitoring and enforcement of the law. Delegations of authority should be accompanied by a commitment to allocate appropriate funds to adequately monitor and enforce the law. Another weakness was that the penalties were often not an adequate enough deterrent, despite laws being accompanied by a broader range of penalties than is found with self-regulatory regimes. Governments must consider the legislative vehicle used to ensure that a wide range of appropriate enforcement provisions and penalties are available under the legislative mandate. For example, injunction powers could require a company to cease using an advertisement campaign in breach of the law, or a power for a statutory body to pre-vet advert campaigns while larger fines or criminal penalties could compel higher compliance.

The main strength of the paper is the application of the conceptual framework that helps to articulate the legislative design components of the three different case study countries, and their approach to regulating the food marketing environment. The key purpose was to interrogate the technical design of each legal approach to aid other governments regulating in a similar space. A similar approach was used by Reeve and Magnusson [18] to review the marketing restrictions of six jurisdictions and Jones et al. to analyse 31 front of pack nutrition labels globally [44]. Similar case study methods have been utilised in relation to other NCD prevention policy interventions in different contexts [79–82].

One weakness of the study is that it seeks to outline the purely technical elements of legislative design but it is acknowledged that the political economy of law and policy making is complex and influenced by political ideologies, power dynamics, and corporate political activity [83, 84].The political nature of legislative design is also an important study that cannot be divorced entirely from a technical investigation of legislation. However, it was decided that this topic requires its own interrogation and therefore is the subject of a forthcoming study that intends to complement this research. This is of most importance, as two of the case studies have failed to be passed into law or implemented, due to political influences on the policy process.

## Conclusion

The WHO and other global health actors have recommended that governments around the world use legislative responses to reduce the exposure of children to all unhealthy food marketing [11, 26, 28, 46].However, governments often lack the technical or legal capacity to undertake the policy design process. The case studies presented in this research provide an insight into legislative design components of not only the substantive content of each approach but the regulatory form and implementation considerations and the governance mechanisms present throughout the process. An analysis of the three laws illustrates an evolution in design over time, as well as the design strengths offered by a legislative approach. However, many opportunities remain for improving legislative responses to protect children from unhealthy food marketing, including extending the scope of legal responses to include brand marketing, sponsorship and earned and owned digital media and to cover the full range of media and settings that children are exposed to, rather than just a select group. Our synthesis and summary of the technical elements of food marketing laws can support governments around the world as they develop their own food marketing policies. Further research is needed to understand the political elements of food marketing laws and what countries can learn from other governments.

## Supplementary Information

**Additional file 1.**

## Data Availability

The datasets generated and analysed during the current study are not publicly available as they are qualitative interview data that is subject to a confidentiality clause in the participant consent forms.

## Abbreviations

NCDs: Non-communicable diseases
WHO Set of Recommendations: World Health Organization Set of Recommendations on the Marketing of Foods and Non-Alcoholic Beverages to Children
UNCRC: United Nations Convention on the Rights of the Child
WCRF: World Cancer Research Fund International
WHO: World Health Organization
UK: United Kingdom
Food Law: Law No. 20.606 on the Nutrient Composition of Food and its Advertising
OFCOM: The Office of Communications
ASA: Advertising Standards Authority
