# Supplementary material for: Designing legislative responses to restrict children’s exposure to unhealthy food and non-alcoholic beverage marketing: a case study analysis of Chile, Canada and the United Kingdom
Source: Global Health. 2022 Jul 23;18:72. doi: 10.1186/s12992-022-00865-x (PMC9308290; doi:10.1186/s12992-022-00865-x)
Supplement: Supplementary file 1 — Additional file 1. [file 12992_2022_865_MOESM1_ESM.docx]

# Online supplementary File

# Methods for documentary analysis

## Full list of Search terms

| Chile AND | marketing AND food; marketing AND food AND barriers; marketing AND food AND policy; marketing AND nutrition AND policy; marketing AND food AND law  advertising AND food; advertising AND food AND barriers; advertising AND food AND policy; advertising AND food AND law  Food and Advertising law; Food ley; Food Law; Food Labelling and Advertising Law; Ley 20869; Ley 20.606, Decree 28, 103 and 13 |
| --- | --- |
| UK OR United Kingdom OR England AND | marketing AND food; marketing AND food AND barriers; marketing AND food AND policy; marketing AND nutrition AND policy; marketing AND food AND law  advertising AND food; advertising AND food AND barriers; advertising AND food AND policy; advertising AND food AND law;  Health and Care Bill; HFSS marketing; Restrictions on HFSS marketing; Restrictions AND HFSS AND price and promotion |
| Canada AND | marketing AND food; marketing AND food AND barriers; marketing AND food AND policy; marketing AND nutrition AND policy; marketing AND food AND law  advertising AND food; advertising AND food AND barriers; advertising AND food AND policy; advertising AND food AND law;  Bill S-228, Nancy Greene Raine AND advertising AND law; Child Health Protection Act; Child Health Protection; M2K |

## Sites searched

Government websites for the three case studies – the relevant Ministry of Health or Department of Health of the country. World Health Organization Regional Offices and Headquarters websites; Google searches for key terms.

## When searches were conducted

Searches were conducted from April 2021 to April 2022.

## Inclusion and exclusion criteria

Inclusion criteria:

- Mentioned the case study policy in question for three countries (Chile – Food and Advertising Law; UK restricting HFSS food and beverage advertising OR Health and Care Bill; Canada: Child Health Protection Bill S228 OR Restricting Unhealthy Food and Beverage Marketing to Children)
- Between the dates relevant for each case (the start of the policy development cycle (Chile: 2006; UK: 2018; Canada: 2015) up until the time of writing the research)
- Any language (as documents were translated using google translate and overarching summary was shown to Chilean key informants to verify accuracy)

Exclusion criteria:

- Documents that discussed a separate policy intervention not related to marketing- for example if the document only discussed the food labelling laws in Canada or Chile which were part of the wider policy strategy.
- Documents outside of the date range specified above.
- Documents about other countries other than Chile, Canada and UK
- Documents about Quebec Region’s policy

## Databases searched for peer-review literature

Scopus, PubMed, Google Scholar, ProQuest, ScienceDirect

## Who made decisions about relevant vs irrelevant material

FS made the decisions about relevant vs irrelevant material
